# Respiratory metagenomics: route to routine service

**DOI:** 10.1097/QCO.0000000000000909

**Published:** 2023-02-01

**Authors:** Jonathan D. Edgeworth

**Affiliations:** Department of Infectious Diseases, Guy's & St Thomas’ NHS Foundation Trust & Department of Infectious Diseases, Kings College London, UK

**Keywords:** community and hospital acquired pneumonia, intensive care unit, pandemic preparedness, respiratory metagenomics

## Abstract

The coronavirus disease 2019 pandemic demonstrated broad utility of pathogen sequencing with rapid methodological progress alongside global distribution of sequencing infrastructure. This review considers implications for now moving clinical metagenomics into routine service, with respiratory metagenomics as the exemplar use-case.

**Recent findings:**

Respiratory metagenomic workflows have completed proof-of-concept, providing organism identification and many genotypic antimicrobial resistance determinants from clinical samples in <6 h. This enables rapid escalation or de-escalation of empiric therapy for patient benefit and reducing selection of antimicrobial resistance, with genomic-typing available in the same time-frame. Attention is now focussed on demonstrating clinical, health-economic, accreditation, and regulatory requirements. More fundamentally, pathogen sequencing challenges the traditional culture-orientated time frame of microbiology laboratories, which through automation and centralisation risks becoming increasingly separated from the clinical setting. It presents an alternative future where infection experts are brought together around a single genetic output in an acute timeframe, aligning the microbiology target operating model with the wider human genomic and digital strategy.

**Summary:**

Pathogen sequencing is a transformational proposition for microbiology laboratories and their infectious diseases, infection control, and public health partners. Healthcare systems that link output from routine clinical metagenomic sequencing, with pandemic and antimicrobial resistance surveillance, will create valuable tools for protecting their population against future infectious diseases threats.

## INTRODUCTION

Clinicians review many sources of information when assessing patients with an acute infectious disease, which for the purposes of this review is on the adult or paediatric intensive care unit (ICU) with a suspected severe lower respiratory tract infection (LRTI). There are multiple viral, bacterial, and fungal pathogens, some with potential for transmission to family, recent community contacts and now healthcare staff, and there may be antimicrobial resistance which complicates treatment choices, particularly if the LRTI was acquired in hospital. There is also the rare but not discountable possibility of novel pathogens including with pandemic potential as happened with Middle East Respiratory Syndrome Coronavirus (MERS-CoV) on our own ICU in 2012 [1] and South Korea in 2015 [2] and with Severe Acute Respiratory Syndrome (SARS)-CoV-2 in 2019 in China [3].

Respiratory and other samples are tested, each for a selected range of pathogens and often in different laboratories that provide results back from within an hour, to a few hours, the next day or many days, and weeks later. Data from biochemistry, haematology, imaging, history, examination, and physiological parameters are available that day, but the identity and antimicrobial resistance (AMR) of causative pathogens are not. Consequently, treatment, infection control and public health decisions are made empirically at different times based on the likelihood and consequence of different pathogens. A total of 25–47% of community LRTI cases remain undiagnosed after all conventional testing [4], and presumably more if certain tests are not requested. Clinicians in every acute hospital in the world deal with this uncertainty every day [5^▪^]. 

**Box 1:**
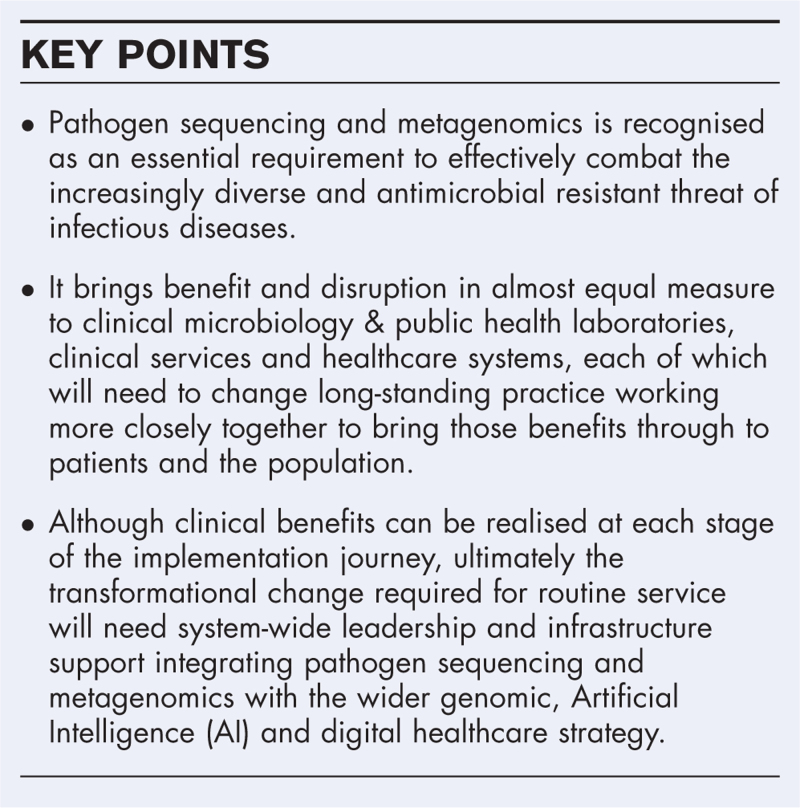
no caption available

## THE RESPIRATORY METAGENOMICS PROPOSITION

The clinical metagenomics proposition in an acute care setting is to sequence nucleic acid from all organisms in a patient sample, to provide all required pathogen information ideally on the day of sample collection. Rapid turnaround time is important but shared by other molecular tests, however it is the comprehensive pathogen-agnostic capability that is the unique aspect setting metagenomics apart from all other tests. The ability to identify conventional agents and their AMR profile, alongside the unexpected, unrequested, hard to detect or novel pathogens, and therefore conversely a more dependable “rule-out” result. Application to respiratory infection has potential for greatest impact, with data from a single test used for patient treatment, antimicrobial stewardship, hospital infection control, public health surveillance and pandemic preparedness [6,7].

**FIGURE 1:**
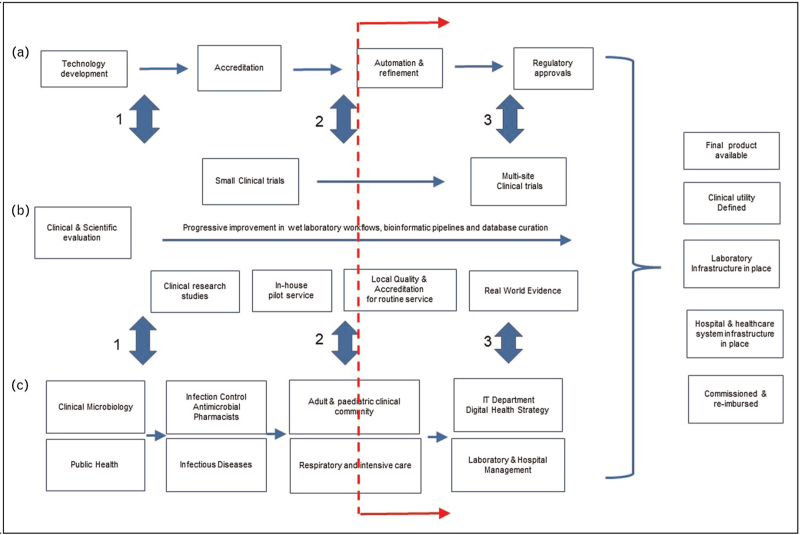
Integrating contribution from different stakeholders to the respiratory metagenomics route to routine service. This is an illustrative perspective from a clinical academic laboratory. Stakeholders will use different terminologies and have different views on ordering and prioritisation of activities which are not necessarily sequential. The core principle is close iterative engagement between each workstream. (a) Industry workstream involves technology development, ISO9001 or ISO13485 accreditation of their products and submission to regulators. (b) Basic, translational and clinical scientist workstream. Involves technology co-development, workflow refinement to optimise performance characteristics, evaluation in small laboratory studies using clinical samples, pilot local service evaluation of internally developed workflows under clinical governance framework, through to ISO15189 accreditation as a laboratory developed test generating real world evidence and involvement in large clinical trials (c) Clinical and service user workstream of all hospital based stakeholders including service providers (microbiology and infectious diseases), infection specialty service users (infection control & antimicrobial stewardship), clinical users (respiratory and intensive care physicians), service enables (IT) and internal decision makers and funders (hospital management). Three main stages of close inter-workstream engagement are represented by vertical filled arrows as (1) Proof-of-concept stage (circa 50–100 sample studies) that a workflow can be safely and reliably performed delivering results that have potential for clinically utility in a required timeframe. This gives support for each workstream to progress to (2) Proof-of-value (circa 500–1000 sample studies) involving early clinical evaluation generating real-world evidence and assessment as a locally developed and accredited test with defined performance characteristics, which informs 3) Proof-of-implementation requiring investment from industry in final product development and regulatory approval, by research funders to support large multisite clinical trials to demonstrate clinical utility and cost-effectiveness and, by healthcare systems implementing infrastructure, producing business cases for using tests, training staff and re-organising clinical pathways to realise benefits. Dotted red line indicates approximate current position.

This opportunity was clearly articulated before the pandemic along with equally matched technical and implementation challenges [8,9], such that progress towards routine service was considered modest. The pandemic changed this balance for many reasons and expedited movement of respiratory metagenomics (RMg) along the translational research pathway (Fig. 1). RMg provided the full SARS-CoV-2 sequence from the first coronavirus disease 2019 (COVID-19) patients [3] and as the pandemic progressed new sequencing capability was placed in infectious diseases laboratories worldwide for SARS-CoV-2 genomic epidemiology. As clinical and academic teams came together through the unifying pandemic imperative [10], the translational research timeline for pathogen sequencing shortened. Diagnostic accuracy studies are beginning to demonstrate satisfactory performance with pathogen detection [11^▪^,12^▪^] and there are innovative approaches to detecting AMR elements using nanopore sequencing with cas9 enrichment [13^▪^] or predicting AMR using machine learning [14]. There is also increasing focus on clinical laboratory quality requirements including validation, reproducibility, setting limits of detection, and producing positive and negative internal controls and externally distributed reference materials for quality assurance [15]. Rapid workflows have been developed [16], evaluated in research setting during the pandemics [17] and taken through into a service setting (Charalampous – manuscript in preparation) using established hospital quality improvement governance processes [18^▪^]. Sequencing companies adapted their offer to infectious diseases, recognising the greater turnaround time imperative compared with human genomic sequencing. Illumina have smaller iSeq 100 and MiniSeq platforms while Oxford Nanopore Technologies have developed the portable Minion with single sample adaptors (Flongle), providing results from end-to-end workflows in <6 h, considered particularly suited for further evaluation in a clinical setting [19]. Detailed characteristics of sequencing platforms have been recently reviewed and not covered here [15,20,21].

With these technical advances have come increasing case reports and series identifying unusual or unexpected pathogens [22–28], comparative evaluations [29–31] and literature reviews [32^▪^,^33^] demonstrating the value of RMg in a real-world adult and paediatric setting. Many were retrospective or observational, but some informed treatment in real time [34] and the protocol for a randomised multicentre clinical trial has been published [35^▪^]. RMg consistently increases identification of causative pathogens, in one paediatric ICU study including noninfective control patients up from 67% with all conventional testing to 92% [36^▪^]. There also remains the need to distinguish infection from colonisation which applies to all microbiological tests. This is particularly relevant for opportunistic infections that require additional investigations and prolonged treatment. RMg provides rapid identification of *Pneumocystis jirovecci* in non-human immunodeficiency virus immunosuppressed patients [37,38], but could lead to unnecessary investigation and overtreatment where significance is uncertain, as might organisms not conventionally detected in the respiratory tract such as *Trophorema whipplei*[39]. These will be important factors to assess in clinical and health-economic studies. Inclusion of the host inflammatory response signature alongside relative abundance of putative pathogens will be a helpful addition [40].

## A ROUTE TO IMPLEMENTATION IN THE HEALTHCARE SYSTEM

**FIGURE 2:**
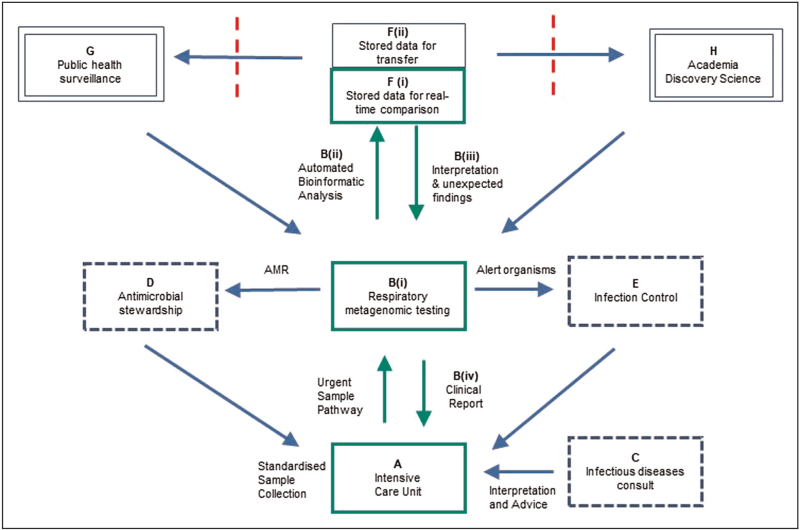
Integrating multidisciplinary working and data flows across the infectious diseases network to realise benefits of respiratory metagenomics. Stakeholder roles and connections currently exist, but information transfer is incomplete and staggered over days, weeks or months. Data is pushed or pulled through different channels in different formats (individual paper or electronic patient reports, spreadsheets, pdf files, phone calls or e mails) with variable interpretation or prioritisation. Transfer often too slow and laborious to meet clinically required time-points so opportunities for prevention, diagnosis or treatment are missed. Lack of common real-time data-visibility frustrates shared learning and prioritisation, and keeps infection professions in silos working cross department, organisation and funding boundaries. A single pathogen genetic data-output simultaneously distributed across the infection network, would support shared real-time assessment and prioritisation. Each professional group identifies components of the common dataset required for continuous service improvement in their domain but with visibility across the network. (a) Standardised sample collection (e.g., BAL or mini-BAL) and targets for laboratory transfer times with sample tracking. (b) includes the [i] Targeted amplicon, pure isolate or metagenomic sequencing from molecular laboratory section or bench. [ii] Automated bioinformatic analysis and interpretation, with [iii] clinical and scientific oversight of unexpected or concerning results to correlate with clinical presentation and alert infection network [iv] comprehensive reports to clinicians during the initial diagnostic evaluation phase. (c) daily infectious diseases or clinical microbiology bed-side or virtual rounds enhanced by including (d) antimicrobial pharmacy and (e) infection control representation. (f) Stored pathogen sequence data, with or without metadata from the electronic health record, improves either i) individual reports potentially with machine learning approaches integrated into real-time report generation (green box) or ii) as a repository for off-line analysis and export. (g) Real-time or intermittent sequence data transfer outside the primary care organisation with information governance approvals (red dashed line) for pathogen surveillance and (h) after anonymisation to academic groups with research governance approval to address knowledge gaps in all aspects of the new infectious diseases framework. Outputs from (g) and (h) fed back to the clinical team.

Given these recent developments this review considers an end-state where every patient having a deep respiratory sample submitted to the diagnostic laboratory has first-line metagenomic testing as standard of care. Culture and targeted molecular tests continue for selected cases or as reflex tests, rather than the other way round, which is the current generally accepted position [21,41]. Targeted testing in selected institutions is an inequitable end-state only partially delivering the benefit of RMg and will be hard to sustain in an acute care pathway dependent on heightened awareness by clinicians and scientists diverting exceptional samples away from the routine sample pathway. This review therefore looks ahead to routine adoption, to consider requirements and potential benefits for clinical teams, laboratory networks and the wider healthcare system (Fig. 2).

### Clinical input to refining metagenomic workflows

Clinical input is needed to define user requirements to develop clear target product profiles that will inform late-stage methodology refinement [42]. It is not straightforward predicting characteristics of a novel molecular test that will ensure uptake into routine service, even by future users themselves. It requires iterative input that can be informed by experience using early prototypes in a pilot setting [18^▪^]. Methods differ in their efficiency extracting nucleic acid from different organisms, given the wide variation in structure and physico-chemical properties of bacterial types, including mycobacteria and those with or without a cell wall, fungi, deoxyribonucleic acid (DNA) or ribonucleic acid (RNA) viruses, protozoa, and other parasites. Some workflows will also retain free DNA from dead organisms, which may or may not be considered clinically useful [43]. Input is required setting clinically significant thresholds for reporting organisms, some of which may differ. For example, thresholds for reporting DNA sequence from a Gram-negative bacteria in a patient with suspected ventilator associated pneumonia could be set at or below the culture threshold. Conversely, no sensitivity limit would be imposed for obligate respiratory pathogens such as *Mycobacterium tuberculosis* or *Chlamydia psittacci.* Indeed, targeted approaches for some respiratory pathogens such as *M. tuberculosis* when their presence is specifically sought [44], which in principle could be incorporated into the metagenomics assay rather than separately. Limitations of different RMg workflows will impact on safe result interpretation, particularly when considering antimicrobial de-escalation based on no (significant) organisms reported.

The content and time of report production needs consideration. Accurate organism identification is possible from sequencing <0.1% of the genome after a few minutes so there may be benefit producing organism profile or “No (significant) organisms detected” reports first [45]. Alternatively, clinicians may prefer waiting for a single report a few hours later that includes presence of specific resistance or virulence genes and predictions for phenotypic susceptibilities. Typing reports from full genome sequence may come later with more detail or full sequence output to infection control, public health and research groups under research ethics framework. These are important considerations because while technological improvement continues apace, clinical workflows need to focus on relevant actionable information, rather than providing everything achievable at the technology limits. Storing large amounts of organism sequence in clinical systems would not be cost-effective, although potentially attractive for academic or surveillance purposes.

### Clinical service and hospital operational requirements

Improving diagnostic pathways will require improvement to the pre-analytic and post-analytic stages in addition to the testing methodology itself. The UK standards for microbiology investigations for blood cultures has set new targets for time to analysis of <4 h alongside bigger blood volumes to increase sensitivity [46]. Similar standards for respiratory samples make no comment on time to analysis other than recording if the sample is not processed on day of collection [47]. International guidelines for hospital and community acquired pneumonia don’t specify turnaround-time either and have different views on quality of sample collection between directed bronchoalveolar lavage samples versus tracheal aspirate [48–50]. This probably reflects the clear link between positive blood culture, rapid sepsis treatment and outcomes [51] that is hard to assess for LRTI when culture is the diagnostic method. RMg can prompt much needed standardisation of turnaround times and quality for the respiratory diagnostic pathway, including for example specifying BALs or less invasive but standardised mini-BAL sampling [52], rather than accepting the heterogeneity associated with tracheal aspirates.

Clinical reporting will need re-evaluation with turnaround times for validation or auto-release rather than batch reporting. At least initially, interpretation may be needed at the bed-side or at daily hybrid MDTs with clinical microbiology, infectious diseases, radiologists, antimicrobial pharmacists and intensivists present. This will be an important opportunity to achieve better consensus about aetiology and diagnosis of LRTI, particularly ventilator-associated pneumonia (VAP), which has suffered from longstanding uncertainty and controversy [53,54] and drives most antimicrobial prescribing on ICU. Scoring systems have attempted to improve VAP diagnosis but are not recommended in guidelines [49], and clinical assessment without supportive microbiology contributes to pneumonia being frequently mis-diagnosed across the emergency pathway [55]. Clinical experience gained by the MDT correlating RMg results with mild or severe infection and noninfective conditions [36^▪^], should improve the understanding, diagnosis and treatment of LRTIs, including the impact of antibiotics on the resistome linked with treatment duration and antimicrobial stewardship decisions [56]. Over time accumulated datasets can be used to develop automated interpretation and decision support, including through machine learning linking metagenomic patterns with diagnosis, severity, treatment, and outcomes

### Quantifying and generating evidence of clinical and health-economic benefit

Providing evidence of combined clinical and health-economic benefits at a system level will be an important driver for RMg adoption and re-imbursement but will likely come from a range of sources and trials that capture the different benefits. They will need to assess (1) Immediate patient benefit, commencing appropriate antimicrobials when organisms are identified predicted to have intrinsic or acquired resistance to empiric therapy. The frequency will depend on prevalence of endemic AMR, local guidelines and individual risk assessment made by attending clinician. (2) AMR benefit, targeting or stopping antimicrobials by identifying organisms predicted to be susceptible to a narrower antimicrobial spectrum or reporting no organisms. Almost all antimicrobial prescribing includes some retrospectively definable over-treatment, so measures of safe achievable reduction are required for clinical trials. (3) Wider patient and organisational benefit through faster identification of organisms with infection control significance prompting interventions to prevent transmission or further cases. (4) Public health benefit, identifying organisms or variants with wider public health significance including increasing community epidemiological trends, new or re-emerging infections.

These benefits will be captured in target product profiles [42] that specify analytical performance alongside clinical validity, utility and cost-effectiveness. Analytical performance studies are relatively straightforward, whereas randomised controlled trials demonstrating clinical endpoints and cost-effectiveness are recognised to be prohibitively expensive for diagnostic tests. They are also conceptually complex because new molecular tests provide information informing individual decisions that are dependent on human beliefs and behaviours [57], as well as the organisations infrastructure and resources to respond. Evidence may need to include real world data [58], virtual patient trial design [59] and proposals for more efficient trial methodologies that assess the realisable clinical benefit of CMg tests will be welcomed.

Finally, there is the challenge with RMg presented as a hypothesis-free test not restricting itself to a predefined list of pathogens, with utility for the unexpected, the un-requested, the unknown or novel agent [6,7]. These are defining features of infectious diseases investigation, and the source of uncertainty that CMg is uniquely positioned to address. Yet this attribute may prove the most challenging for accrediting and regulatory bodies. Their standards are becoming more demanding with ISO15189:2012, soon to be updated, new accreditation requirements for machine learning components of a diagnostic test with SO14971:2022, and regulatory approvals in some jurisdictions in a transition period moving from the In Vitro Diagnostic Device (IVDD)-Directive to more demanding IVDD-Regulations. Their early and iterative involvement in defining an achievable framework for gathering required evidence is preferable.

### Laboratory operational and healthcare system strategic requirements

Most pathogen sequencing is currently performed in academic or public health laboratories but will need to be performed in diagnostic laboratories for routine service. Indeed, there may be advantages moving the dominant sequence capability into the clinical laboratory [60], with data rather than occasional samples transferred to academic and public health laboratories for further analysis (Fig. 2). It would increase the speed and volume of samples sequenced, reduce laborious send-away processes and duplication, and place the first sequence evaluation with staff linked with the presenting case, to optimise co-assessment of pathogen genomics with detailed clinical phenotypes.

Establishing pathogen sequencing within the microbiology laboratory has implications for the target operating model, which in many countries is for consolidation including to off-site facilities, utilising increased automation of culture-based core laboratory workflows. This is anticipated to enhance investment in molecular diagnostics and innovation, while improving quality and turnaround times [61]; however ideally not at the expense of further separating the infection discipline into patient-facing and laboratory-facing components. Pathogen sequencing and metagenomics could unify these requirements, providing comprehensive rapid results in a clinically relevant timeframe, while integrating data output from across a healthcare network for AMR and infection control surveillance benefits.

Every laboratory network will have different factors to consider in placing sequencing capability alongside relevant clinical and scientific expertise, ensuring efficient sample transport, clinical reporting and data infrastructure. For some it may be considered a disruptive additional change overlaid on existing plans. For all, it will require investment that is probably beyond the diagnostic laboratory budget or strategic remit and will need leadership from the healthcare system embedding infectious diseases within its wider genomic, artificial intelligence, and digital strategy [62,63]. It certainly shares with cancer and human genetics the transformative opportunity of personalised medicine alongside the infrastructure, ethical and information governance requirements analysing, storing and transferring genetic data particularly across organisational boundaries. Paradoxically, countries with a less mature existing microbiology infrastructure, may be able to leap-frog the current laboratory model, moving pathogen sequencing faster and more efficiently into their future healthcare system, unrestrained from compromising around the traditional operating model [64].

## CONCLUSION

This review summarises the dramatic progress in pathogen sequencing during the COVID-19 pandemic, which served as a reminder that serious emerging and re-emerging infections are frequent events, exacerbated by global warming, biodiversity loss, international trade and travel [64]. When combined with increasing AMR and diversity of opportunistic pathogens in the context of immunosuppression, the need to modernise microbiology services by routine provision of pathogen sequencing is recognised as an even more urgent priority [65]. RMg is moving to the forefront of the pathogen sequencing agenda, based on its ability to link routine testing for personalised patient antimicrobial treatment with improvements in infection control, AMR and public health surveillance and ultimately the societal response to new pandemic threats. Further technology development and laboratory assessment is certainly required transitioning from early clinical evaluation to routine diagnostic provision, but the focus now is more on gathering evidence that the clinical and wider healthcare opportunities of RMg and other pathogen sequencing tests can be cost-effectively realised upon implementation into healthcare systems. Perhaps more importantly, given the transformational change required, is the need for healthcare systems to begin integrating pathogen sequencing alongside cancer genetics and human genomics as part of their wider transformation strategy.

## Acknowledgements

*None.*

### Financial support and sponsorship

*JE received funding from the National Institute for Health Research (NIHR) Biomedical Research Centre based at Guy's and STtThomas’ National Health Service (NHS) Foundation Trust and Kings College London, the programme of Infection and Immunity (RJ112/N027) and the Guy's and St Thomas’ Fund grant TCF 190910. JE receives research funding from Oxford Nanopore Technologies under a collaboration agreement with Guy's & St Thomas’ Hospital.*

### Conflicts of interest

*JE is employed part-time by Oxford Nanopore Technologies*

## REFERENCES AND RECOMMENDED READING

Papers of particular interest, published within the annual period of review, have been highlighted as:

▪ of special interest
▪▪ of outstanding interest
